# Deep learning: To better understand how human activities affect the value of ecosystem services—A case study of Nanjing

**DOI:** 10.1371/journal.pone.0238789

**Published:** 2020-10-06

**Authors:** Chang Liu, Yi Qi, Zhenbo Wang, Junlan Yu, Shan Li, Hong Yao, Tianhua Ni

**Affiliations:** 1 School of Geography and Ocean Science, Nanjing University, Nanjing, China; 2 School of Architecture and Urban Planning, Nanjing University, Nanjing, China; 3 Institute of Geographic Sciences and Natural Resources Research, Chinese Academy of Sciences, Beijing, China; 4 School of Geography, Nantong University, Nantong, China; Institute for Advanced Sustainability Studies, GERMANY

## Abstract

The value of ecosystem services is affected by increasing human activities. However, the anthropogenic driving mechanisms of ecosystem services are poorly understood. Here, we established a deep learning model to approximate the ecosystem service value (ESV) of Nanjing City using 23 socioeconomic factors. A multi-view analysis was then conducted on feasible impact mechanisms using model disassembly. The results indicated that certain factors had their own significant and independent effects on ESV, such as the proportion of water areas in the land-use structure and the output value of the secondary industry. The proportion of ecological water should be increased as much as possible, whereas the output value of the secondary industry should be reasonably controlled in Nanjing. Other intrinsically related factors were likely to be composited together to affect ESV, such as industrial water consumption and industrial electricity consumption. In Nanjing, simultaneously optimizing socio-economic factors related to city size, resources, and energy use efficiency likely represents an effective management strategy for maintaining and enhancing regional ecological service capabilities. The results of this work suggest that deep learning is an effective method of deepening studies on the prediction of ESV trends and human-driven mechanisms.

## Introduction

Ecosystem services are the benefits that people obtain from various ecosystems that can be described and evaluated [1, 2], and they exist in the form of provisions (e.g., timber and water), support (e.g., nutrient cycling), regulation (e.g., carbon sequestration), or cultural activities (e.g., recreation and spiritual uses) [3]. Mendelsohn and Olmstead described the values of ecosystem services (ESVs) as “the sum of what all members of society would be willing to pay” for “the economic benefit provided by environmental products or service” [4]. This definition reflects one of the basic theories of accessing ecosystem services, which is an individual preference and willingness to pay. Another basic theory is utility value theory, which demonstrates that ecological products have the characteristic of scarcity and can be useful to people similar to general social commodities [1]. This analogy also reveals that the maximization of ESVs in a region may be as important as economic development [5]. Hence, the estimation of ESVs can make a vital contribution to ecological protection and sustainable development [6, 7]. ESV assessments at the national, regional, basin, and even single ecosystem levels can show how these services support our lives and how we utilize natural resources and ecological products [7, 8]. The valuation methods now available are highly developed and can be mainly divided into behavioral (revealed preference) methods and attitudinal (stated preference) methods [4]. Behavioral methods attempt to calculate the environmental value of goods indirectly through market analysis [9–11]. Attitudinal methods use subjectively designed surveys to create a table of ecological value equivalents. Two common valuation systems include the system created by Costanza in 1997 and the millennium ecosystem assessment framework [1, 12].

However, the understanding of ESVs is not comprehensive because multiple types of services interrelate in complex and dynamic ways [13–15]. The current research perspectives on ESVs consider these services to be the result of a process: “human-driven factors of ecosystem change → ecosystem processes and functions → ecosystem services” [13]. “Human-driven factors of ecosystem change” can be interpreted as basic socioeconomic conditions, including population, Gross Domestic Product (GDP), industry structure, and energy consumption. “Ecosystem processes and functions” can be represented by land and land cover change at the geospatial level, which is traditionally the most important part of the information used to estimate ESVs [13]. However, the interaction between ESVs and socioeconomic factors remains ambiguous [16–19], which leads to difficulties in the application of ESVs in ecological management [17]. In other words, even if a sensitive area with irreversible declining ESVs is identified, we still do not know how to reduce the loss of the value of ecosystem services efficiently through regional planning or industry regulation. Studies have begun to include the socioeconomic drivers of ESVs into consideration for the implementation of responsive policies. Yang used Pearson’s correlations to examine the relationships among ecosystem services and found that ESVs are tightly correlated with socioeconomic status in the Beijing-Tianjin-Hebei Metropolitan Area [20]. Wu found nonlinear relations between GDP and ESV and a similar pattern between population density and ESV by employing analyses of variance (ANOVA), although a more complete causality was not explained [21]. Chen used GeoDetector and principal component analysis (PCA) to discuss the drivers of the spatial distribution of ESV in Beijing, and the results indicated that in areas where human activities are highly concentrated, the ESV distribution is strongly disturbed by the population density and GDP [22]. In addition, Li showed an inverted U-shaped coupling trend between ESV and population density, which meant that ESV will rise initially and fall later with increases of population [23]. However, this study did not identify the specific inflection point of the population density value that can be used as a reference for regional planning and management. In general, most existing studies on human-driven mechanisms of socio-economic factors on ESVs focus on the traditional phase and only conduct qualitative descriptions or extract relatively important socioeconomic factors using statistical analysis methods, such as correlation analysis, regression analysis, GeoDetector, PCA, etc. [20–24].

As a type of machine learning algorithm, deep learning constructs hierarchical architectures of increasing sophistication, and artificial neural networks with many perceive layers are examples of deep learning algorithms [25]. Deep learning offers significant breakthroughs in solving classification and nonlinear regression problems [26] and can extract the valid features of data input through complex computational models and represent them at a higher level of abstraction, eventually achieving complex self-learning functions through multiple transformations and combinations [27]. Traditional evaluation and analysis methods are often insufficiently effective in describing the continuous and quantitative rules in a complicated ecosystem [28]. Deep learning may be an effective tool for dealing with this problem.

In this work, deep learning was used to explore the relationships between “human drivers of ecosystem change” and “ESV” on a dataset from Nanjing City, China. The model for predicting ESV by inputting socioeconomic factors was established, and it was disassembled to analyze the quantitative relationship between socioeconomic factors and ESV. Based on the research results, corresponding specific policy recommendations were proposed for realistic regional ecological environmental protection and resource management.

## Materials and methods

### Study area

**Fig 1:**
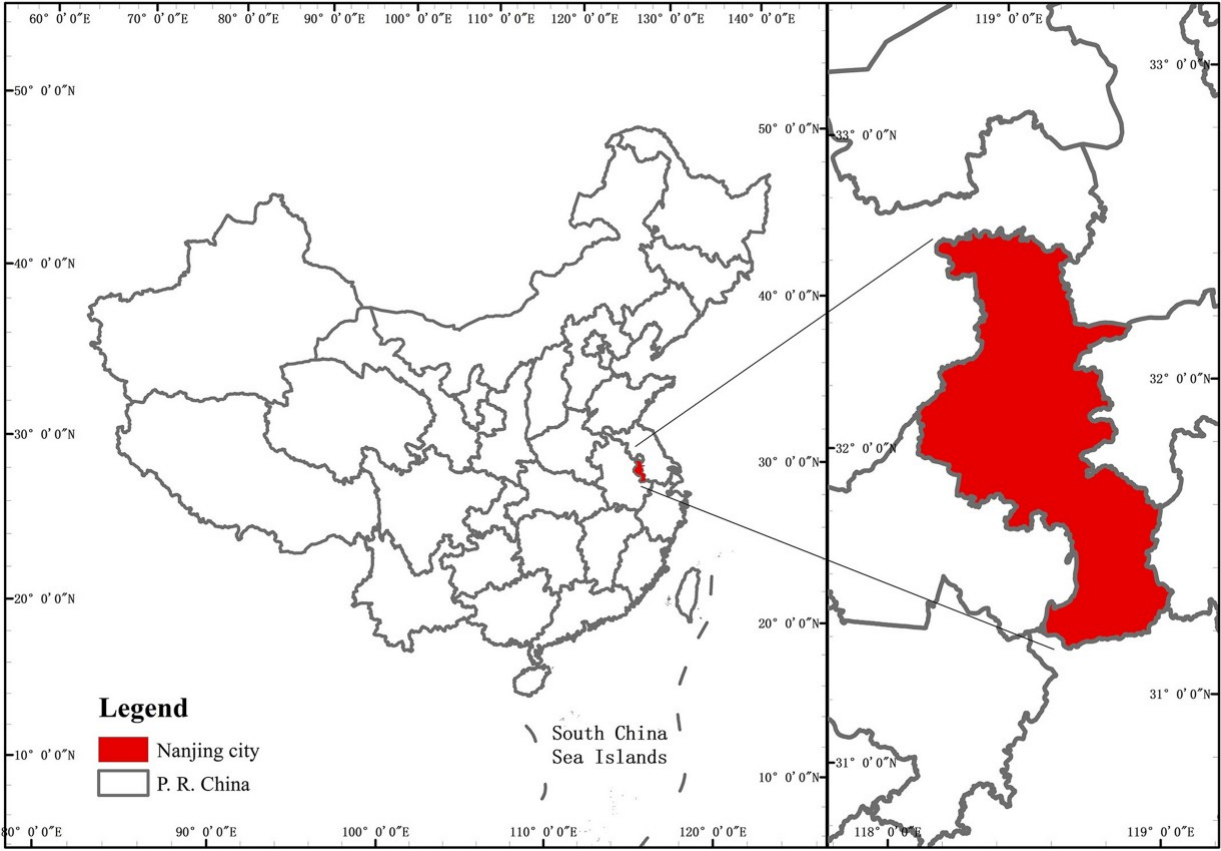
Location of Nanjing city.

Nanjing (31°14″-32°37″ N, 118°22″-119°14″ E) is the capital city of Jiangsu Province and has a total area of 6597 km^2^ (Fig 1). This city is one of the megacities in the Yangtze River basin and has experienced rapid economic development since the 1970s that is still occurring today [29]. At the end of the 20th century, the urbanization of Nanjing entered an accelerated phase, which led to the rapid increase in population, unreasonable industrial structure, unbalanced land use, high energy consumption, and environmental degradation [30, 31]. Over the last two decades, the population has increased from 3 M to 8.5 M, and its GDP has increased from 338.12 billion CNY in 2008 to 1171.51 billion CNY in 2018. As an ecologically sensitive area, the changes in its ecological system and services have been continuously monitored and studied. Taking ESV as an ecological parameter that generalizes the state of interaction between the regional ecosystem and human socioeconomic system, a better understanding of the internal driving mechanisms will be conducive to optimizing local policies and regional planning [32].

### Data preprocessing

The Terrestrial Ecosystem Service Value Distribution Database used in this study was constructed by Xie using the equivalent factor method, which assumes that each unit of land area is a functional unit that provides ecosystem services and products [5] (Table 1). The equivalent coefficients table was modified based on a direct comparison with the average global ESV valued by Constanza in 2003. In addition, the contingent valuation method was introduced as expert knowledge using a questionnaire for ecological researchers to ensure that the coefficients were consistent with Chinese ecosystem conditions. This ESV dataset contains the value of eleven types of ecosystem services that can be divided into four primary categories: provisioning services (food production, materials production, and water supply), regulating services (gas regulation, climate regulation, hydrology regulation, and environmental purification), supporting services (soil conservation, nutrient cycling, and biodiversity), and cultural services (aesthetic landscape). The sum of all ESV types was used as the model output, which can reflect the overall ecological conditions and sustainable development level.

**Table 1:** Inputted index system and data sources.

Criteria layer	Code	Index layer	Data sources	Date type and spatial revolution
Output: Y		Ecosystem service value (CNY)	https://doi.org/10.12078/2018060503	Raster/1*1 km
Growth quality (Input: X)	A1	GDP (million yuan)	http://doi.org/10.12078/2017121102	Raster/1*1 km
	A2	Population (person)	https://doi.org/10.12078/2017121101	Raster/1*1 km
	A3	Light index	http://www.resdc.cn/data.aspx?DATAID=213	Raster/1*1 km
	A4	1st industry output value (million CNY)	http://221.226.86.104/file/2017/index.htm	Statistical data
	A5	2nd industry output value (million CNY)		
	A6	3rd industry output value (million CNY)		
	A7	Construction industry output value (million CNY)		
	A8	Tourism output value (million CNY)		
Ecological protection (Input: X)	B1	NDVI (Normalized Difference Vegetation Index)	https://doi/org/10.12078/2018060601	Raster/1*1 km
	B2	Cultivated area (m^2^)	http://www.resdc.cn/data.aspx?DATAID=184	
	B3	Woodland area (m^2^)		
	B4	Grassland (m^2^)		
	B5	Water area (m^2^)		
	B6	Construction land area (m^2^)		
	B7	Unused land area (m^2^)		
Resource utilization (Input: X)	C1	Electrical consumption (kw)	http://221.226.86.104/file/2016/index.htm	Statistical data
	C2	Agricultural electricity consumption (kw)		
	C3	Industrial electricity consumption (kw)		
	C4	Residential electricity consumption (kw)		
	C5	Water consumption (kt)		
	C6	Agricultural water consumption (kt)		
	C7	Industrial water consumption (kt)		
	C8	residential water consumption (kt)		

According to China’s green development policy [33], the factors related to ecological conditions and sustainability were divided based on six aspects: resource utilization, environmental governance, environmental quality, ecological protection, growth quality, and green life. Due to the spatial scale of this study, natural conditions, such as temperature, perception, and humidity, are relatively similar across the city; therefore, they were not used as model input for differentiation. Taking the correlations with ESVs, anthropogenic controllability, and data accessibility into account, 23 indexes from 2015 were chosen and altered from three perspectives (Table 1) as input for the following ESV deep learning model. Among these indexes, the “Light Index” is a factor that can reflect the urbanization process and economic development level and the “NDVI” (Normalized Difference Vegetation Index) represents the regional vegetation coverage and plant growth. The data resources and spatial revolution of raster data are shown in Table 1, and the source of all spatial data is the open-source Resource and Environment Data Cloud Platform (http://www.resdc.cn) built by the Chinese Academy of Science (CAS) [34–37].

Multidata fusion on the same scale is necessary to ensure that the labeled samples meet the common format and quantity requirements for deep learning model training [38–40]. The area of Nanjing is approximately 6500 km^2^. Because ESVs are influenced by a multiplicity of socio-economic and natural factors, if the selected spatial unit is too small, then demonstrating the composite effect will be difficult. Moreover, model training and testing have a minimum requirement for the sample size. Hence, we used a 2*2 km grid which is better than 1*1 km and 5*5 km grids for data processing by grid transformation. A total of 2191 grid units were obtained as samples.

To spatialize the socioeconomic data, the population, land use, and light index were used as weighting factors for allocation under limited data and technical support. Because a strong correlation likely occurs between the land use structure and industrial structure, different land-use scenarios probably play different roles in influencing the development of urban industries [41]. For example, agriculture contributes more than 60% of Nanjing’s 1^st^ industry output and agricultural products are also employed as raw materials by the livestock husbandry and fishery industries. Therefore, we chose the cultivated area as the weighting factor of the allocation of 1^st^ industry output value. Because the tourism industry is closely related to urban development, the light index, which can represent regional prosperity, was selected as a weighting factor. Energy consumption is related to population and land use type [42]; therefore, the corresponding indexes were selected, such as the industrial electricity/water consumption-industrial land area, residential electricity/water consumption-residential land area. According to the key weighting factors (Table 2), socioeconomic data were allocated using Eq 1, and spatial socioeconomic data with a resolution of 2*2 km were obtained.

$$Af_{i}=As_{i}\times \frac{wf_{i}}{{\mathrm{ws}}_{i}}$$ (1)

where Af_i_ represents the values in 2*2 km units of the ith (No.) allocated indicators; As_i_ represents the total value of the ith (No.) allocated indicators in Nanjing City; Wf_i_ represents the values in 2*2 km units of the ith (No.) weighting factors; and Ws_i_ represents the total value of the ith (No.) weighting factors in Nanjing City.

**Table 2:** Socioeconomic data and the corresponding weighting factors.

Code	Allocated indicators	Weighting factors
**A4**	1^st^ industry output value (million CNY)	Cultivated area (m^2^)
**A5**	2^nd^ industry output value (million CNY)	Industrial land area (m^2^)
**A6**	3^rd^ industry output value (million CNY)	Residential land area (m^2^)
**A7**	Construction industry output value (million CNY)	Construction land area (m^2^)
**A8**	Tourism output value (million CNY)	Light index
**C1**	Electrical consumption (kw)	Population (person)
**C2**	Agricultural electricity consumption (kw)	Cultivated area (m^2^)
**C3**	Industrial electricity consumption (kw)	Industrial land area (m^2^)
**C4**	Residential electricity consumption (kw)	Residential land area (m^2^)
**C5**	Water consumption (kt)	Population (person)
**C6**	Agricultural water consumption (kt)	Cultivated area (m^2^)
**C7**	Industrial water consumption (kt)	Industrial land area (m^2^)
**C8**	Residential water consumption (kt)	Residential land area (m^2^)

Finally, data standardization was conducted as a standard procedure before training. Standard deviation standardization was used to eliminate the influence of the magnitudes. The land-use factors were represented by individual area proportions (from 0% to 100%) for each type, which were then individually standardized.

### Data modeling

Because of the complexity of the anthropogenic driving mechanisms, the more essential and quantifiable relationships of the 23 independent variables for ESVs are difficult to describe with conventional models [5]. We chose Multilayer Perception (MLP) as the deep learning model, which is also called the Multilayer Feedforward Dense Network. It is a generic nonlinear function approximation algorithm that has been extensively used for problems such as function approximation, prediction, and classification. It is the most widely used type of network because of its flexibility and simple structure, which are also beneficial to the subsequent model analysis.

The network consists of 8 layers (4 dense layers), including 6 hidden layers in the network. Each layer has a certain number of neurons and activation functions (Table 3). Nonlinear activation functions, such as the rectified linear unit (ReLU), were introduced in the 3^rd^ and 5^th^ hidden layers to learn the nonlinearity. The ReLU function was used to avoid vanishing gradient problems. Additionally, the dropout rate was set to 0.3 in all dropout layers to avoid overfitting problems.

**Table 3:** Configuration of the model.

Layer	Operation	Parameter	
**Input layer**		23 Socioeconomic factors	
**1, 2-hidden**	dense	128 neurons	linear
	dropout	0.3	
**3, 4-hidden**	dense	256 neurons	tanh
	dropout	0.3	
**5, 6-hidden**	dense	16 neurons	ReLU
	dropout	0.3	
**Output layer**	dense	1 neuron	linear

We partitioned 70% of the 2191 units as training samples and 30% as testing samples. In the training phase, the optimizer and loss function were established based on the adaptive moment estimation (ADAM) and mean square error (MSE). After conventional model optimizations were performed, the above hyperparameters were determined. The corresponding model was trained and used in the study.

### Model analysis

We observed how ESV (Y) responded to the change in each influence factor (X) by sampling continuously in the range of each input X. In the course of the concrete analysis, other factors were kept as the mean of the samples and the values of the target factors were changed by adopting control variables. Also, the range of target X was regarded as its definition domain, called the sampling domain. The ESV range varies in terms of the target X in its sampling domain, which is called the response domain. The influence intensity of every X factor can be judged according to the corresponding response domain, and the influence mode and potential mechanism can be judged according to the variation trend of the function.

## Results and discussion

### Model performance

**Fig 2:**
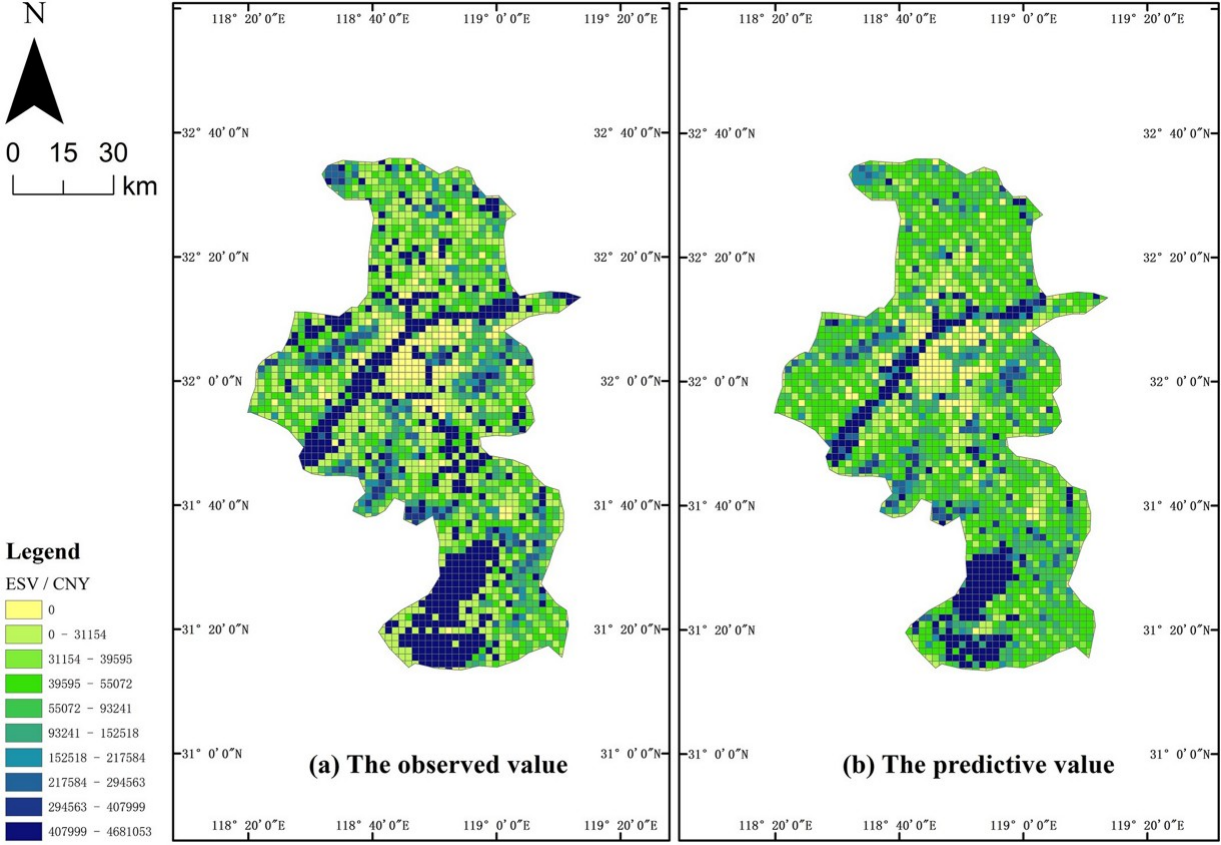
Comparison map of the observed ESV and predictive ESV in Nanjing. (a) The observed value; and (b) the predictive value.

The model was trained for 200 epochs and showed a significant convergence trend. In terms of precision, the Nash-Sutcliffe efficiency and root MSE (RMSE)-observation standard deviation ratio (RSR) were used as indicators to reflect the model performance, and they reached values of 0.51 and 0.70 respectively, indicating “satisfactory” performance. Spatially, the observed value and predictive value of each sample were visualized with one set of legends showing similar spatial characteristics (Fig 2). However, overall, the sum of the predictive values was lower than the ground truth sum.

### Single-factor response

Twenty-three X factors were divided into three categories according to the range of the response domain: extremely significant influence intensity (ES), significant influence intensity (S), and nonsignificant influence intensity (NS) (Table 4). The threshold values were set as 0.2 and 0.1. The index was ES when its range of response domain was higher than 0.2; the index was S when its range of response domain was higher than 0.1 and lower than 0.2; and the index was NS when its range of response domain was lower than 0.1.

**Table 4:** Influence intensity and influence modes of 23 X factors.

Index	Original sampling domain	Standardized sampling domain	Range of response domain	Function shape
2^nd^ industry output value (million yuan)	(0, 14000)	(0, 14)	0.310 (ES)	monotone decreasing
Water area (%)	(0, 100)	(0, 100)	0.236 (ES)	monotone increasing
GDP (million yuan)	(0, 10000)	(0, 15)	0.181 (S)	monotone increasing
Industrial water consumption	(0, 40)	(0, 14)	0.178 (S)	monotone decreasing
Light index	(-100, 600)	(-2, 5)	0.162 (S)	increasing with fluctuation
3^rd^ industry output value (million yuan)	(0, 1750)	(-1, 5)	0.154 (S)	monotone decreasing
Agricultural electricity consumption	(0, 0.35)	(-2, 3)	0.138 (S)	monotone decreasing
Water consumption (kt)	(0, 70)	(0, 20)	0.138 (S)	inverted U-shaped
Tourism output (million yuan)	(0, 80)	(-6, 1)	0.137 (S)	increasing with fluctuation
1^st^ industry output value (million yuan)	(0, 35)	(-2, 3)	0.13 (S)	monotone decreasing
Woodland area (%)	(0, 100)	(0, 100)	0.103 (S)	monotone increasing
Grassland area (%)	(0, 100)	(0, 100)	0.094 (NS)	U-shaped
Residential electricity consumption (kw)	(0, 20)	(-1, 5)	0.089 (NS)	monotone increasing
NDVI	(0, 4)	(-3, 3)	0.089 (NS)	U-shaped
Population (person)	(0, 250000)	(0, 17.5)	0.088 (NS)	U-shaped
Agricultural water consumption (kt)	(0, 2.5)	(-2, 3)	0.083 (NS)	U-shaped
Electrical consumption (kw)	(0, 800)	(0, 20)	0.079 (NS)	inverted U-shaped
Construction industry output (million yuan)	(0, 175)	(-1, 5)	0.071 (NS)	inverted U-shaped
Industrial electricity consumption (kw)	(0, 1000)	(0, 14)	0.067 (NS)	inverted U-shaped
Residential water consumption (kt)	(0, 20)	(-1, 5)	0.051 (NS)	U-shaped
Construction land area (%)	(0, 100)	(0, 100)	0.042 (NS)	monotone decreasing
Unused land area (%)	(0, 100)	(0, 100)	0.037 (NS)	monotone increasing
Cultivated area (%)	(0, 100)	(0, 100)	0.017 (NS)	monotone decreasing

**Fig 3:**
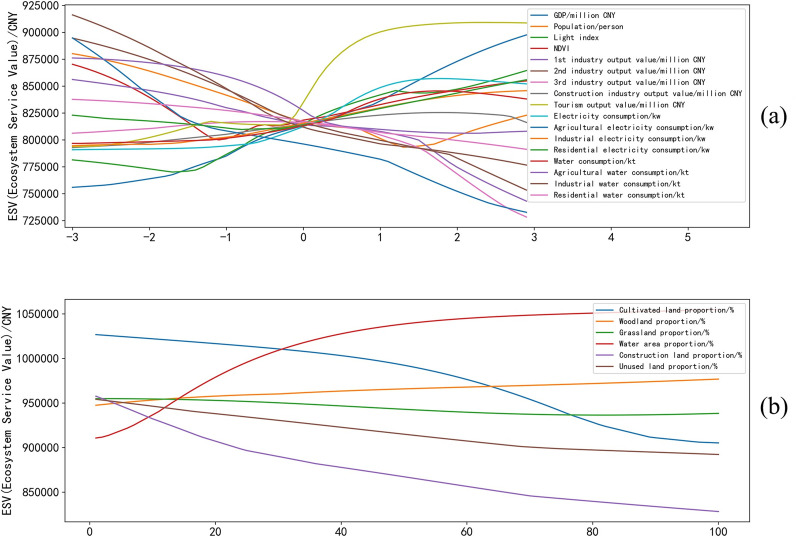
Functions of the relationships between standardized X factors and ESV. (a) Function of the relationship between nonland-use-type factors (changed from -3 to 3) and ESV; and (b) function of the relationship between land-use factors (changed from 0% to 100%) and ESV.

**Fig 4:**
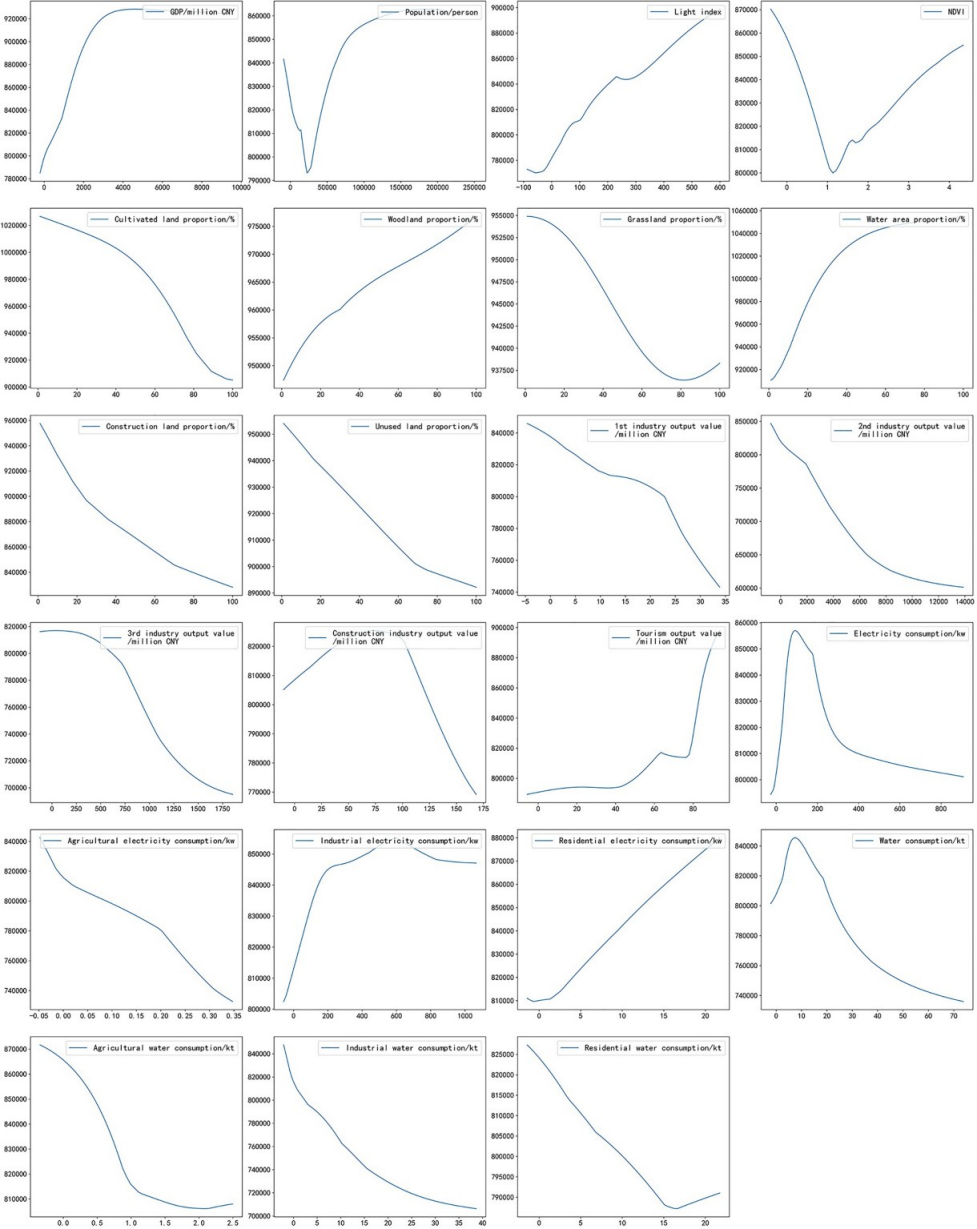
Functions of the relationships between X factors before standardization and ESV.

The “2^nd^ industry output value” was an ES factor with the highest range in the response domain. The monotonic decreasing function of the “2^nd^ industry output value” and ESV meant that a more developed 2^nd^ industry led to a lower ESV (Fig 3A). In addition, there were a series of X factors with a relatively high range of response domains and significant influence intensity. Among them, “GDP”, “light index” and “tourism output” had a positive impact on ESV and “industrial water consumption”, “3^rd^ industry output value”, “agricultural electricity consumption” and “1^st^ industry output value” had a negative impact on ESV. The function of the relationship between “water consumption” and ESV adopted an inverted U-shaped curve. The inflection point of “water consumption” was approximately 10 kt, while ESV reached a maximum value of 810 k (Fig 4). This result suggested the possibility of the existence of a reasonable interval of water consumption in Nanjing.

“Water area” was another ES factor, and the areas of other types of land were all NS factors except “woodland area”, which was S and displayed the lowest range in the response domain compared to other S factors. The “water area”, “woodland area” and “unused land area” had a positive impact on ESV (Table 4). In contrast, the “cultivated area” and “construction land area” had a negative impact on ESV. Moreover, the relationship between “grassland area” and ESV displayed a U-shaped curve that had an inflection point at approximately 80% of the grassland area (Figs 3B and 4).

Other factors had a relatively low range of response domains, which meant that their influence was NS. However, almost all the functions capturing the relationship between these NS factors and ESV assumed a U-shaped curve or an inverted U-shaped curve (Figs 3B and 4). All the analyses suggested that there is a response relationship between ESV and socioeconomic conditions. Therefore, ESV can be improved indirectly by adjusting these factors. However, understanding how to regulate and control the above factors to achieve a balance between ESV and economic development still requires further multifactor coupling research.

Compared with existing research, some of our results are controversial or contrary, such as the function of relationships between ESV and the GDP, light index, and population values. Yu found that the ESVs of China was decreased slightly while the GDP value was increased substantially [43]. However, Xie found that there is no linear relationship between the total ESV and GDP [5]. Li found that the ESV in cities of Shannxi Province increased slightly or remained stable with the growth of GDP [44]. However, all these studies did not control variables, which means that the relationship between ESVs and GDP also includes the impact of other socioeconomic factors on ESVs. In addition, the single-factor response of our model reveals the driving law of single-factor variance to ESV. We argue that the socio-economic drivers of ecosystem services are likely to be different for cities with different sizes, in different regions, and even at different stages of development. If the relationship between GDP and ESVs assumes a U-shaped curve, then different laws may be obtained due to different selection of study areas. Thus, Nanjing City may be at a later stage of development and thus will fall on the right side of the U-shaped curve. Similarly, the relationships among population, light index, and ESVs are also controversial [45–48] For example, Han found that the influence trend of the population on ESV is different in the high-ESV region and low-ESV region [45]. Studies on the relationships between ESVs and urbanization generally indicate that urban expansion has positive or negative correlations with the values of different ecosystem services [47, 48].

Moreover, certain limitations are observed with this model. The absence of significant input variables may lead to inaccurate mechanism interpretations. In future research, changes in model analysis results may be observed by changing the structure and input variables to screen the most reasonable interpretation model.

### Multifactor response

Achieving the maximum ESV and adjusting all socioeconomic factors to the optimum intervals are difficult because of the complex coupling influence. We attempted to select several sets of factors with significant interactions to identify which proportions could regulate the factors for the purpose of maximizing ESV. As a result, the pairs “the proportion of construction” and “population”, “1^st^ industry output value” and “population”, and “industrial water consumption” and “industrial electric consumption” were chosen to analyze how they cooperatively influence ESV.

**Fig 5:**
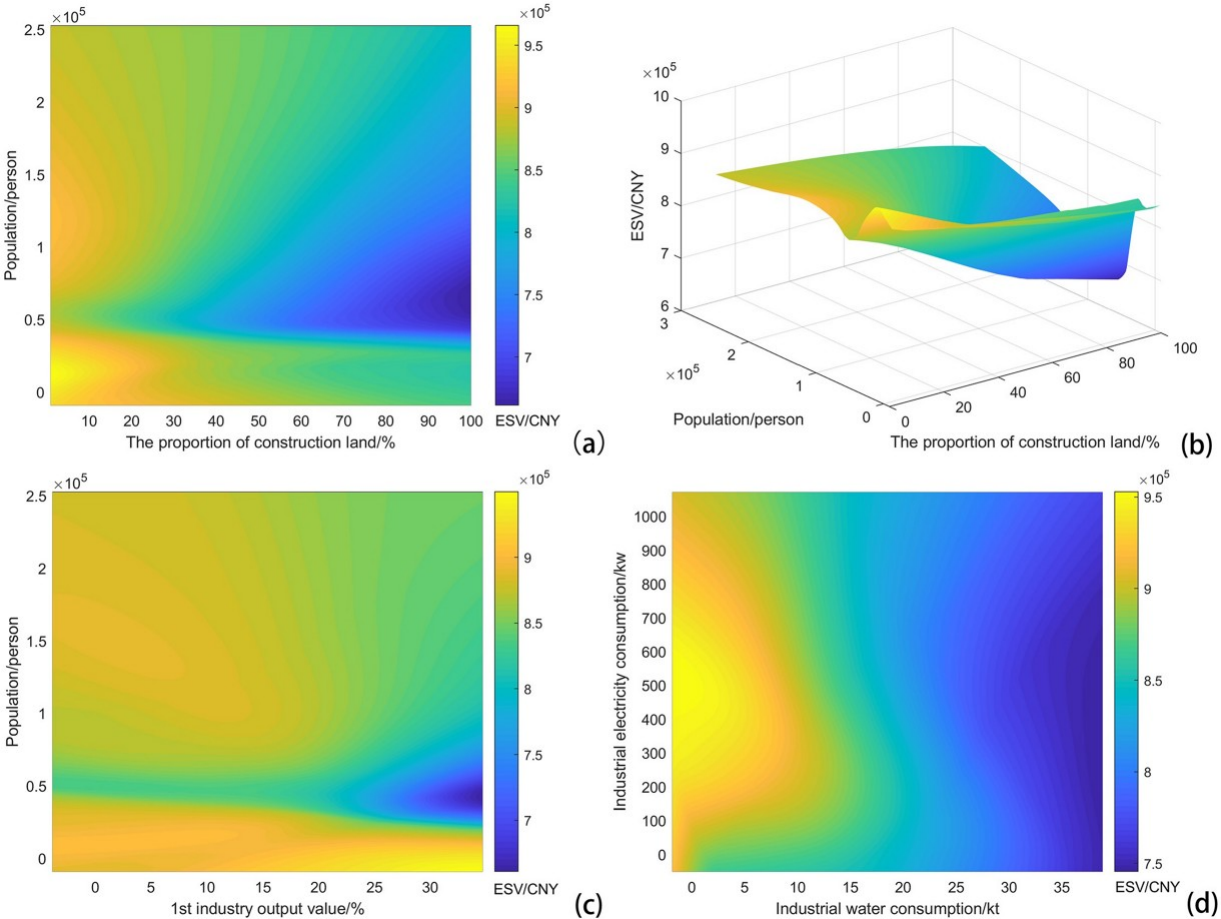
Surface of ESV and three sets of coupling factors. (a) Coupling influence of “the proportion of construction” and “population”; (b) 3D surface of ESV under “the proportion of construction”, and “Population”; (c) coupling influence of “1^st^ industry output value” and “population”; and (d) coupling influence of “industrial water consumption” and “industrial electric consumption”.

This approach suggested that ESV is most affected by population (Fig 5A). The population within 4 km^2^ should be controlled to less than 20 k or approximately 120 k. In addition, ESV dropped sharply with increases in construction land when the population was 50 k. In contrast, ESV declined slowly with the increase in construction land when the population was more than 100 k, which suggests that a relatively single distribution of construction land will not place too much pressure on the ecosystem in densely populated centers. However, controlling construction land still has a positive influence on ESV. Therefore, the city center can sacrifice nonconstruction land for infrastructure construction and population accommodation and should reserve ecological space.

The coupling influence of “1^st^ industry output value” and “population” showed that there were two scenarios that achieve higher ESV (Fig 5B). One scenario was when the population was nearly 0 and the output of the 1^st^ industry was more than 15 million CNY. Under this scenario, the object region had a small population but played an important role in agricultural production. The other scenario was when the population was approximately 150 k and the 1^st^ industry output value was less than 10 million CNY.

Regarding energy consumption, “industrial water consumption” was presumably the limiting factor compared with “industrial electricity consumption” (Fig 5C). Industrial water consumption should be kept below 15 kt if one hopes to achieve a high ESV. When industrial electricity consumption was between 400 and 600 kw, ESV rapidly decreased with increasing industrial water consumption. This finding suggested that water consumption needs to be restricted and reserved in industrial areas.

### Urban cognizable synergistic features

Because the activation function in the output layer is linear, cognitive and comprehensible features can be extracted from the penultimate layer [49]. Nine features with bottom-up information were interpreted and understood. Considering the range of Y and the weight of the features, significant features that contained more information about the corresponding factors were selected. The features were named according to X, which had a great influence on them except feature 7, which contained information on almost all the factors, and features 9 and 15, which contained little information on any factors. Therefore, 6 cognizable synergistic features integrated from multiple factors were extracted, including the urban expansion factor (0.0133), land use-industrial structure-energy consumption structure (0.0146), land use-energy consumption structure, agricultural development (0.0146), city-scale factor (0.0151) and tourism exploitation potential (0.0138) (Table 5). Among these factors, the city-scale factor was the most significant urban cognizable feature with the highest weight (0.0151), and it contained information about GDP, population, and tourism output. This finding demonstrated that the deep learning model may predict ESVs by quantifying regional characteristics, including land-use structure, energy consumption structure, industrial structure, and city scale. Therefore, it is possible to adjust the urban macro characteristics to maintain or even improve the regional ESVs.

**Table 5:** Extraction and analysis of urban cognizable features.

Number	Weight	Factors contained	Weighted range of Y	Urban cognizable features
Feature 1	0.0133	Light index	0.000508	Urban expansion factor
		Cultivated area	0.000768	
		Residential electricity consumption	0.000365	
Feature 3	0.0146	Light index	0.00083	Land use-industrial structure-energy consumption structure factor
		Cultivated area	0.000902	
		Woodland area	0.00108	
		Grassland area	0.000862	
		Water area	0.001034	
		Unused land area	0.000916	
		2^nd^ industry output value	0.000484	
		3^rd^ industry output value	0.000488	
		Agricultural electricity consumption	0.000358	
		Industrial water consumption	0.000469	
		Residential water consumption	0.000543	
Feature 6	0.0146	Woodland area	0.00106	Dynamic land use-energy consumption structure factor
		Water area	0.001037	
		Construction land area	0.000803	
		Unused land area	0.001005	
		Construction industry output value	0.000309	
		Electrical consumption	0.000628	
		Industrial electricity consumption	0.000926	
		Residential electricity consumption	0.000424	
		Water consumption	0.000347	
		Agricultural water consumption	0.000369	
Feature 8	0.0143	Light index	0.000645	Agricultural development factor
		Cultivated area	0.000905	
		Unused land area	0.000978	
		1^st^ industry output value	0.000547	
		Agricultural electricity consumption	0.00036	
		Residential electricity consumption	0.000439	
		Water consumption	0.000379	
Feature 10	0.0151	GDP	0.000964	City scale factor
		Population	0.001185	
Feature 13	0.0138	Population	0.000885	Tourism exploitation potential factor
		NDVI	0.000846	
		Agricultural water consumption	0.000819	
		Tourism output value	0.000632	
		Construction land area	0.000376	

The extraction of urban cognizable synergistic features could be regarded as a form of dimensionality reduction among the 23 X factors. Therefore, we compared urban cognizable features with the results of a principal component analysis, which is a widely used technique in machine learning [50]. Six principal components were identified, and they all contained no more than 2 factors (Table 6). However, we could not provide a definite practical meaning for the principal components, which meant that the extraction of urban cognizable features has unique advantages as a new dimensionality reduction method.

**Table 6:** Results of the principal component analysis.

Principal component number	Factors contained	Eigenvalue
1	GDP	0.97
	Population	0.24
2	GDP	-0.23
	Population	0.95
	Tourism output value	-0.2
3	Population	-0.19
	Tourism output value	-0.98
4	Light index	0.99
5	2nd industry output value	0.99
6	Construction land area	0.19
	3rd industry output value	0.97
	Residential electricity consumption	0.11

## Conclusions

This study proposed deep learning as a new more effective approach to understanding the patterns, dynamics, and driving factors of ESVs, which are crucial for coping with sustainability challenges. The findings of the model analysis suggested that underlying social and economic conditions presumably influence regional ecological functions through ESVs.

Regarding Nanjing City, although the outputs of the 1^st^, 2^nd^, and 3^rd^ industries all showed a decreasing trend in ESV, the “2^nd^ industry output value” had the highest influence intensity, indicating the urgency and necessity of controlling its proportion. We propose that economic development, urbanization, and tourism should be further accelerated and enhanced in Nanjing because “GDP”, “light index”, “tourism output” and “residential electricity consumption” all have positive influences on ESV. In addition, there should be exclusivity in the urban function, which means that city space needs to be separated to serve different functions. The extraction of high-level urban cognizable factors related to ESV in the penultimate layer may be a new dimensionality reduction method, and the analysis suggested that the city scale of Nanjing can truly affect the ESV. As a result, decision-makers can provide policy guidance and adjust urban features to realize the coordinated development of the regional economy and ecological functions. For instance, the most suitable city scale can be found that is within the regional ecological carrying capacity.

This research focused on the relationship between human socioeconomic development and ESVs on the urban scale. We built a deep learning model based on limited socioeconomic factors, extracted cognizable synergistic factors and obtained meaningful results. Furthermore, we suggest that obvious differences likely occur in the driving mechanisms under diverse regional and scale contexts. Therefore, an interesting direction for further research is investigating more influence patterns and mechanisms on diverse spatial scales and levels of socioeconomic development that affect the change in different regional ESVs. In addition, differences may occur among the driving mechanisms of multiple ecosystem services, such as supporting services, provisioning services, regulating services, and cultural services. Therefore, our method and concepts could be used to analyze ESV characteristics and driving mechanisms to understand these differences.
